# Sexual dysfunctions, internalized stigma and quality of life in patients with schizophrenia

**DOI:** 10.1192/j.eurpsy.2023.337

**Published:** 2023-07-19

**Authors:** A. BEJAR, N. SMAOUI, I. GASSARA, R. FEKI, S. OMRI, M. MAALEJ BOUALI, N. CHARFI, J. BEN THABET, L. ZOUARI, M. MAALEJ

**Affiliations:** psychiatry “C”, Hedi Chaker university hospital, Sfax, Tunisia

## Abstract

**Introduction:**

Schizophrenia is a chronic psychotic disorder characterized by a high prevalence of sexual dysfunctions (SD). SD can affect the quality of life (QOL) of patients, cause low self-esteem and self-stigma.

**Objectives:**

To evaluate the sexual functioning, the QOL, and the internalized stigma among outpatients with schizophrenia.

To determine the links between SD, the QOL, and the internalized stigma.

**Methods:**

A cross-sectional, analytical study was conducted between Mars and September 2019. It included 53 outpatients with schizophrenia in clinical remission for at least two months.

We used the Arizona Sexual Experiences Scale (ASEX) to assess sexual functioning, the Internalized Stigma of Mental Illness scale (ISMI) to assess the subjective experience of stigma, and the 36-item Short-Form Health Survey (SF-36) to evaluate the QOL.

**Results:**

The average age of patients was 42.28 years old, and their sex ratio was 3.81. The average ASEX score was 19.77±5.99, and 67.9% of participants had at least one SD.

The mean ISMI score was 2.47±0,34. 60,4% of patients had a high level of internalized stigma. The QOL was impaired in 66% of the cases.

We found correlations between SD and a high level of internalized stigma (p=0.011) and its subscales «alienation » (p=0.013), «stereotype endorsement» (p=0.034) and « discrimination experience» (p =0.001).

SD correlated with impaired QOL (p<0.001), emotional limitation (0.050), and social functioning (0.031).

**Conclusions:**

Our study highlights the importance of the impact of SD on the prognosis of schizophrenia through internalized stigma and altered QOL.

**Disclosure of Interest:**

None Declared

